# Cognitive Rehabilitation in Patients with Gliomas and Other Brain Tumors: State of the Art

**DOI:** 10.1155/2016/3041824

**Published:** 2016-07-14

**Authors:** E. Bergo, G. Lombardi, A. Pambuku, A. Della Puppa, L. Bellu, D. D'Avella, V. Zagonel

**Affiliations:** ^1^Department of Clinical and Experimental Oncology, Medical Oncology 1 Unit, Veneto Institute of Oncology IOV-IRCCS, 35128 Padua, Italy; ^2^Neurosurgery Department, Azienda Ospedaliera di Padova, Padua, Italy; ^3^Neurosurgery Department, University of Padua, Padua, Italy

## Abstract

Disease prognosis is very poor in patients with brain tumors. Cognitive deficits due to disease or due to its treatment have an important weight on the quality of life of patients and caregivers. Studies often take into account quality of life as a fundamental element in the management of disease and interventions have been developed for cognitive rehabilitation of neuropsychological deficits with the aim of improving the quality of life and daily-life autonomy of patients. In this literature review, we will consider the published studies of cognitive rehabilitation over the past 20 years.

## 1. Introduction

Brain tumors represent 2% of the total incidence of cancer. They can occur at any age but they are more common in the elderly. The primary adult tumors include meningiomas, schwannomas, primary CNS lymphomas, and gliomas of the cerebral hemispheres (particularly glioblastoma multiforme, anaplastic astrocytoma and, more benign, low-grade astrocytoma, and oligodendroglioma).

High-grade gliomas (HGG), WHO grade III or grade IV [1, 2], are the most common primary brain tumors in adults and glioblastoma multiforme (GBM) is the most frequent glioma in this population. Median overall survival (OS) in patients with GBM remains poor, 15 months for newly diagnosed GBM [3, 4] and 5–7 months for recurrent/relapsed GBM. The overall incidence of brain tumor is virtually the same in males and females, but glioblastoma multiforme is more frequent in men, while meningiomas and schwannomas occur more often in women.

Given the poor prognosis of many brain tumors, the primary objectives of the therapy are to reduce morbidity and restore or preserve neurologic functions and the capacity to perform daily activities as long as possible.

It is widely accepted that brain tumors and related treatments can impair cognitive function across many domains and can have an impact on patients' quality of life. Cognitive deficits in patients with brain tumors can be caused by the tumor itself, by tumor-related epilepsy, by treatment (surgery, radiotherapy, antiepileptics, chemotherapy, or corticosteroids), and by psychological distress [5]. Cognitive deficits are a frequent symptom in patients with brain tumor.

In 139 patients with brain tumor, Tucha et al. [6] found that about 90% of patients displayed impairments in at least one area of cognition. Impairment of executive functions was observed for 78% of patients, and impairment of memory and attention was observed for more than 60% of patients. A recent study by Zucchella et al. [7] showed that, in 147 patients with brain tumor, 54.4% (80 patients) showed cognitive impairment: 43 (53.75%) presented multidomain impairment, while 37 (46.25%) revealed cognitive deficits limited to language (*n* = 13, 16.25%), memory (*n* = 11, 13.75%), attention (*n* = 7, 8.75%), logical-executive functions (*n* = 5, 6.25%), and visuospatial abilities (*n* = 1, 1.25%).

A recent review of the literature by Gehrke et al. [8] indicated that the most common cognitive disturbances cited in the existing research are deficits in memory (working memory), executive functions (cognitive control and flexibility, cognitive processing speed, visual searching, planning, and foresight), and general attention, above and beyond the effects of age, education, and gender.

Cognitive rehabilitation in glioma patients may have an important role achieving both an improvement in neurocognitive functions and a better quality of life after treatment [5]. Cognitive rehabilitation efforts have proven effective in patients with stroke [9, 10] and Alzheimer's disease [11] but only few studies have analyzed the potential benefits of such rehabilitation for patients with primitive brain tumor and even fewer studies have evaluated its impact.

Studies assessing results of cognitive rehabilitation in brain tumor have appeared only recently in the literature.

Cognitive remediation/rehabilitation therapy is based on the principles of neural plasticity of the brain. It is a type of rehabilitation that offers exercises aimed at improving various domains of cognition such as attention, memory, language, and executive/control functions. The expected result is an indirect positive impact on functional deficits affecting everyday life. Proper treatment with these therapies can help enhance the social and professional integration of patients.

Two main types of exercises are used for this treatment: compensation and retraining. In retraining, we retrain brain functions with regularly repeated exercises specific for the deficient aspect of a cognitive function. This is referred to as restoring the deficient function. Another rehabilitation technique is to work with the preserved cognitive functions by compensation techniques. In this case, the patient will be encouraged to develop alternative means to achieve goals. New strategies can include the use of planners, checklists, or memory notebooks. These two forms of training work together: restorative processes help to develop compensatory strategies and vice versa. The final goal is to increase functionality of patients [12].

The purpose of this concise literature review is to analyze the research carried out with the specific aim of rehabilitating cognitive function in patients with cerebral tumor.

We also wanted to ascertain whether any real benefit is associated with these neuropsychological training interventions and the difficulties related to the specific research protocols in this field.

## 2. Methods

### 2.1. Data Sources

An online literature search was performed in PubMed, Medline, and Web of Science for articles published between January 1995 and December 2015. In addition, the reference list of all identified publications was checked. The following search terms were used: brain tumor, glioma, cognitive therapy, exercise therapy, cognitive rehabilitation; cognitive remediation therapy, and neurocognitive training.

### 2.2. Study Selection

Studies were included if all of the following criteria were met: adult patients aged ≥18, primitive brain tumors including gliomas, and studies written in English. Randomized control trials, clinical trials, observational trials, and retrospective trials were included. In total, 42 articles were found, 6 were excluded because they were duplicates, and 21 because they were either congress abstracts or were not written in English.

**Figure 1:**
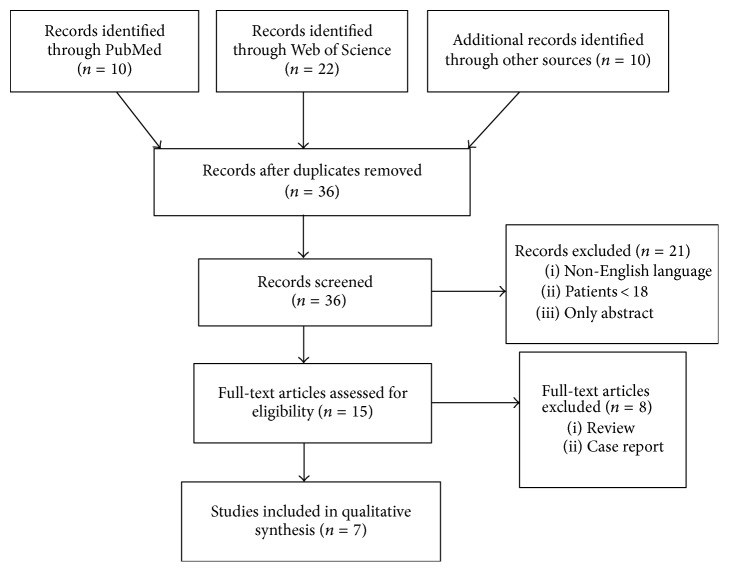
PRISMA flow diagram of article selection.

We found 15 publications regarding cognitive rehabilitation (Figure 1); 7 were excluded, as they were reviews of the literature and one because it was a case report. Thus, seven were reviewed: 4 randomized control trials (RCT) [14, 15, 17, 18], one clinical trial (CT) [16], one retrospective trial (RT) [19], and one observational study (OS) [13].

## 3. Study Characteristics

### 3.1. Population Studied

The study population includes patients with primary brain tumor and, in particular, patients with high-grade glioma (HGG) and low-grade glioma (LGG) and their caregivers in nonpharmacological cognitive rehabilitative intervention studies. One publication included both patients and caregivers [18] and two publications included both patients with primary brain tumor and patients with brain metastasis [13, 14]. Population characteristics of the studies considered are reported in Table 1.

### 3.2. Cognitive Evaluation and Time of Assessment

Taking into account the instruments used for the assessment of cognitive functions before and after training, we can see that, in selected studies, various data collection methodologies are employed (see Table 2). If we take into consideration the domain of function tested, we can see that studies look at several of the same functions.

Yang et al. [14] investigated with a computerized neuropsychological test (CNT) [51] functions of continuous concentration on visual and auditory items, selective attention, verbal and spatial memory, and visual-motor coordination. At baseline, patients were also evaluated with the Korean version of MMSE (K-MMSE) [32] and the Korean version of Barthel index (K-MBI) [31] to assess activities of daily living. Patients were assessed before treatment and after 4 weeks of rehabilitation training.

In their study, Zucchella et al. [15] proposed a neuropsychological evaluation that included a primary screening on patients' language deficit. Patients without aphasia were evaluated for global cognitive functioning, verbal and spatial immediate memory span, verbal memory, immediate and delayed recall, nonverbal reasoning, frontal functionality, simple speed processing, complex attention, visual selective attention, visuoconstructional abilities, and verbal fluency. Cognitive functions of patients were assessed at baseline (T0) and after 4 weeks (T1).

Gehring et al. [17] evaluated patients before starting training with Medical Outcomes Study (MOS) and Cognitive Functioning Scale (CFS) to test presence of self-reported cognitive symptoms. Then, patients who were enrolled for training were assessed with a battery of neuropsychological tests administered at baseline, after cognitive rehabilitation (the control group was evaluated at equivalent time point of training group), and six months after treatment. This particular battery can be administered at hospital or at home. The aim of researchers in using this battery was to evaluate attention, verbal memory, executive functions, motivations, and general cognitive functions.

In the study by Locke et al. [18], researchers wanted to evaluate the impact of cognitive rehabilitation on patient quality of life and emotional distress. They also wanted to investigate the impact that this training can have on caregivers and the degree of appreciation of the program. Patients were assessed with a comprehensive neuropsychological battery at baseline and at 2 weeks and at three months of follow-up (Table 2). Functions assessed were immediate memory, visuoconstruction abilities, language, attention, and delayed memory. Patients also completed the Compensation Techniques Questionnaire to determine compensation techniques used before and after training periods. At the end of the training, they also completed a program satisfaction survey. Overall quality of life, caregiver quality of life, mood, and fatigue were also assessed.

Hassler et al. [16] assessed verbal memory, attention, visual-motor speed, executive functions, and verbal fluency in patients at pre- and posttraining with a battery with 4 tests. Posttraining evaluation was made at 12 weeks. The aim of the study was to evaluate whether rehabilitation of cognitive functioning was possible in patients with glioma and whether it would help patients to restore abilities in daily life.

In the observational study carried out by Maschio et al. [13], patients were assessed at baseline, after cognitive rehabilitation (T1), and after 6 months (T2) with a comprehensive battery of neuropsychological tests (see Table 2). Neuropsychological assessment wanted to investigate global neurocognitive performance, attention, executive functions, abstract reasoning, visuospatial abilities, long-term visuospatial memory, short-term and long-term auditory-verbal memory, and language.

Sherer et al. [19] retrospectively investigated a sample of 13 patients with primary malignant brain tumor, assessing cognitive functions by neuropsychological tests. Domains evaluated were verbal and visual memory, intellectual, executive functions, language, motor, visual perception, and mood. Also, productivity status and level of independence were assessed. Cognitive assessment was made at admission, discharge, and follow-up. No indication about tests used for admission was given.

### 3.3. Program of Rehabilitation

Also, with regard to the rehabilitation training programs used in the studies taken into account, we can find various types of training programs administered. Each program also included a different number of training sessions and follow-up sessions (Table 3).

The rehabilitation program used by Yang et al. [14] used virtual reality (VR) to improve cognitive functions. This type of training, based on real-time and three-dimensional environment, allows patients to gradually confront some aspects of daily living ability. The aim of the computer-assisted rehabilitation program was to improve memory and attention skills. Patients in the intervention group received the virtual reality program and computer-assisted cognitive rehabilitation together. For these patients, VR training was done 3 times a week for 30 minutes and computer cognitive rehabilitation 2 times a week for 30 minutes. Patients in the control group only received computer-assisted cognitive rehabilitation 5 times a week for 30 minutes. Each group was trained for a total of 4 weeks. Functions that improved by this program were memory (spatial memory, recognition, sequential recall, verbal recall, verbal categorization, and verbal and nonverbal memory), attention (discrimination, visual perception, auditory perception, continuous attention, integration, and emotional attention), and daily living skills.

In Zucchella et al.'s [15] program of rehabilitation, the study wanted to investigate the effect of early neurocognitive rehabilitation in patients with primary brain tumor after surgical resection (early cognitive training can improve neuropsychological performance?). Training was administered in one-hour sessions, four sessions per week, for 4 weeks (16 sessions in total). Each session included computer exercises guided by a neuropsychologist. Exercises trained different cognitive functions. Kinds of software utilized for computerized exercises were “training di riabilitazione cognitiva” [48] and “una palestra per la mente” [49]. Both groups received usual medical and physiotherapy rehabilitative care. Domains that improved in rehabilitation training were time and spatial orientation, visual attention, logical reasoning, memory, and executive functions.

The study by Gehring et al. [17] aimed to investigate whether a multidomain cognitive rehabilitation program can have a real effect on objective and subjective evaluations of cognitive functions in patients with glioma. Patients were divided into two groups: intervention group and a control group (waiting list group). The intervention group received cognitive training in two-hour sessions for six weeks. The intervention consisted in cognitive retraining and compensation techniques based, in particular, on rehabilitation of attention. Compensation training consisted of six psychoeducational sessions that included both practical and tutorial lessons targeted at improving memory, attention, and executive function. Regarding retraining, a specific computer program developed by the researchers (Concentration Car, C-Car) [50] was used. This program focused on attention retraining. In addition, patients were asked to complete the computerized homework. Three months after the end of the training, patients received a follow-up session by telephone aimed at strengthening certain aspects of the compensation training. Patients in the control group received usual care only and no cognitive rehabilitation training. At the end of the intervention group's training, the control group also received rehabilitation training. The program wanted to improve attention, memory, and executive function.

In the study by Locke et al. [18], the rehabilitation program used provided both cognitive rehabilitation and problem-solving therapy. Patients were divided into two groups: intervention and control group. The intervention group received training based on six sessions of both cognitive rehabilitation and problem-solving therapy, over two weeks. The control group received standard medical care and no rehabilitation. In the cognitive rehabilitation program, patients and caregivers learned to use a memory notebook for compensation of memory deficits. In the problem-solving program, patients and caregivers learned a positive problem-solving model useful in everyday life. Functions that improved in the training program were memory, attention, and problem-solving skills.

Regarding the Hassler et al. [16] study, the rehabilitation program comprised of one session a week for 10 weeks. Each session lasted 90 minutes. The program used in this study was the holistic mnemonic training program developed by Dr. Stengel. This training comprised the use of all the senses, emotions, and intellect of patients in exercises designed to develop skills in everyday life. Each session addressed, separately, all aspects of mental activity. Perception, concentration, attention, memory, retentiveness, verbal memory, and creativity were the domains rehabilitated.

Maschio et al.'s [13] study aimed to evaluate the effect of cognitive rehabilitation in patients with epilepsy and cognitive disease related to primary brain tumor. The rehabilitation program was provided once a week for ten weeks. TNP software (neuropsychological training software) [37, 38] was the program used for training. The training program aimed to stimulate the residual abilities of the patient to allow maximum autonomy. Training exercises included word and image lists, selection and recognition of targeted stimuli, spatial orientation, and phonological abilities. Domains trained were memory, attention, visuospatial functions, language, and reasoning.

In Sherer et al. [19], the goal of the rehabilitation therapy was individual for each patient: return to work or increased community independence or return to school. The timing of rehabilitation program was individualized for each patient and aimed at decreasing the impact of patients' functional impairments. The typical session day lasted 5 hours. Once a patient improved his/her functions at hospital, he/she was transitioned to an occupational setting therapy where he/she could perform skills similar to the desired vocational goal. At the end of the program, patients were helped to return to their desired productive activities.

## 4. Results

Yang et al. [14], in intervention group, found statistically significant improvements in visual and auditory continuous performance scores, both digit span tests, both visual span tests, verbal and visual learning tests, and TMT A scores. Control group demonstrated an improvement in auditory continuous performance score, visual and verbal learning tests, and forward visual span test. MMSE tests showed improvement in both groups. It seems that VR helps patients by increasing motivation and results are improvement in attention and short-term memory. Both groups improved in autonomous activities of daily living.

In the study by Zucchella et al. [15], at the beginning, no differences in neuropsychological measurements among groups were found. At the end of the rehabilitation training, the study group showed significant improvement in all neuropsychological tests. Control group also had a trend improvement but it was not statistically significant. Therefore, the study group performed better than the control group in all domains. However, a statistically significant difference was only found in visual attention and verbal memory but not in logical-executive functioning.

Gehring et al. [17] found that, at baseline evaluation, the two groups showed no differences in neuropsychological tests but only in MFI scores and SF-36 MCS (worse in intervention group). Over time, significant differences were observed between groups in objective evaluation of cognitive functions, in particular, on attention and verbal memory. In subjective evaluation, differences were found in CFS total score, CFQ total score, and burden. Shortly after the end of the training, researchers found no significant differences between groups in neuropsychological outcome. However, 6 months after the end of the treatment, they found that scores of the study group differed significantly from control group for attention and verbal memory. Regarding subjective outcomes, they found that, shortly after the end of training, the intervention group had better reported cognitive functioning, but at 6-month evaluation, no differences were found. At this time point, only mental fatigue was different between groups (better in study group). Patients reported that this type of training was very useful and strategies learned during the rehabilitation period were also used in everyday life. A decrease in everyday impact of cognitive deficits was shown in 79% of patients treated.

In the study by Locke et al. [18], at the end of the training, evaluation of compensation techniques used by patients demonstrated that 88% of patients in study group used the study-specific strategies in the range of several times a week (minimum) to several times a day (maximum). At three-month follow-up, patients using techniques in the range of several times a week (minimum) to several times a day (maximum) were 50%. Eighty-eight percent employed them but less frequently in time. As for satisfaction of the training program, 88% of patients and caregivers think that the program could be “very helpful” or “somewhat helpful.” Unfortunately, it was not possible to know if the assessed cognitive functions had improved, remained stable, or deteriorated over time, because many of the patients did not show up for the follow-up evaluation.

In Hessler et al. [16], the evaluation of neurocognitive functions done before and after training demonstrated that an enhancement across all neurocognitive functions assessed was achieved. This improvement was statistically significant only in total learning scores of HVLT test. All patients were very satisfied with the training.

Maschio et al.'s [13] cognitive assessment after rehabilitation training showed improved scores in span forward, long-term visuospatial memory, episodic memory, and phonetic fluency compared to the baseline. The same functions remained stable at 6-month follow-up.

Results of the study by Sherer et al. [19] demonstrate that patients improved during the treatment period and that this gain was generally maintained at follow-up 8 months after discharge. Patients had increased community, employment and financial independence, and quality of life. Also, caregiver burden was decreased (Table 4).

## 5. Discussion

Intact cognitive function is necessary for patients with brain tumor to have good quality of survival and independence in everyday life.

Usually, cognitive rehabilitation is rarely offered to patients with brain tumor because they are not seen as potential clients due to their poor prognosis.

Rehabilitation training typically used with patients with brain tumors is borrowed from studies of cognitive rehabilitation in patients with other acquired brain injuries. It is possible that rehabilitation training used in patients with other brain injuries has not had the same results in patients with brain tumor. This type of patients has different cognitive deficits because they usually have no site specific deficits (e.g., stroke patients) but more global cognitive deficits.

A methodological limit of some of the studies considered is that they did not have a control group [13, 16, 19]. Probably, this is one of the main limitations found in these studies because without a control group it is not possible to determine whether improvements made by patients are really due to training or are due to spontaneous recovery of some functions.

Among the studies taken into account, none included a placebo group. This would be the best condition of study to be taken into account. A study without a placebo group cannot state with certainty that the changes of the study group are only due to the effect of training [21, 39].

In most studies, however, to include a placebo group is considered unethical and impractical.

One way to cope with this problem is to create a control group that does not initially benefit from the treatment, being inserted onto the so-called waiting list as Gehring et al. [17] did in their study. In fact, in this study, patients in the control group had the opportunity to participate in the training to rehabilitation until at the end of the study.

Another problem was the different type of brain tumors analyzed in the studies. For example, in the largest and very important study of Gehring et al. [17], there were reported 117 patients with diagnosis of LGG and 23 with HGG; in the other randomized study by Zucchella et al. [15], in 58 patients, 25 had HGG, 7 had LGG, and all other patients had nonglioma tumors. Yang et al. [14] reported 5 GBM and 2 astrocytomas and the remaining patients had nonglioma tumors. In Locke et al. [18], 13 patients had HGG and 6 LGG. Even so, the majority of patients were diagnosed with glioma.

Another critical point is that these patients were studied at different times during the treatment: one study [15] enrolled postneurosurgical patients within two weeks after surgery; other studies enrolled patients in a period where they are clinically stable without therapy [16, 17] and another study during radiotherapy or chemotherapy [13]; lastly, one study analyzed patients during the first recurrence of the disease [18]. It then becomes difficult to understand if any interference in the results of the various studies is due to the treatments received or not received.

In the studies taken into account for this review, we have seen that different tests were used to assess cognitive functions. In a noteworthy retrospective study [19], the tests used were not mentioned in the manuscript.

Yet, the training programs used in the various studies were different and not comparable. Indeed, some authors principally used computer programs [13–15], while others used psychoeducational or ecological techniques [16, 18, 19] or a mix of the previous techniques [17].

This implies that even though they measured the same domains of specific functions, it cannot always be possible to compare the results of various tests, because they measure different specific aspects of a particular cognitive domain.

It is not clear what the best timing of assessment and training is and what intensity should be used. To date, no guidelines exist indicating when and in what kind of patients an intervention with cognitive rehabilitation may be useful. The study by Gehring et al. [17] has a training strategy lasting six weeks, with weekly two-hour meetings; the study by Zucchella et al. [15] used a shorter period of time (four weeks) but with more sessions (four) per week. Similarly, Yang et al. [14] used a 4-week period with shorter sessions a week. Conversely, the training proposed by Locke et al. [18] provides four length sessions similar to those of Gehring et al. [17] but repeated several times during one week and only for two weeks. In other nonrandomized studies [13, 16], longer training periods (10 weeks) in single weekly sessions were preferred.

It seems to be important to train patients early before surgery. In this period, cognitive reserve of patients can be used to try to recover cognitive functions before starting other treatments [15]. Another study, however, shows that patients do not improve immediately after the end of the treatment but they need more time to be able to integrate new strategies learned in everyday life [17].

As reported by various studies, the residual cognitive deficits have a major impact on perceived quality of life of patients. Considering this, it is understandable how important it is to also include in the evaluation of patients a detection of the quality of life at the beginning and the end of the treatment, to see if a real positive effect was achieved also from this point of view.

Unfortunately, not all studies considered QOL assessment [17, 18].

IDH1 and IDH2 gene status and MGMT gene promoter methylation could be important in order to program neurocognitive rehabilitation. Indeed, those genes are important prognostic factors in glioma patients; in particular, IDH1 and IDH2 can be mutated in low-grade and anaplastic glioma, as well; in fact, mutations of IDH1 and IDH2 genes are found in 70% of grades II and III gliomas and secondary glioblastomas [1]. Patients with IDH mutations have a longer survival than patients with IDH wild type. Unfortunately, no studies analyzed these important prognostic factors to determine their impact on neurocognitive rehabilitation program.

Recently, various studies showed that, in addition to the different types of treatments carried out, a number of specific molecular features linked to cancer may affect the presence of cognitive deficits in patients with brain tumors [23]. Investigated genes are involved in various functions such as neuronal repair pathway (ApoE gene), neurotransmitter pathway (COMT), DNA repair, inflammation, and metabolism.

Regarding ApoE gene, Correa et al. [25] found that ɛ4 carriers had worsening in neurocognitive performance. Authors concluded that ApoE polymorphisms may increase vulnerability of patients with brain tumor to cognitive dysfunctions related to treatments. In another study, Correa et al. [30] analyzed 150 patients with brain tumors assessed with neuropsychological tests and showed that COMT gene polymorphisms are associated with performance in working memory, attention, and executive functions. Furthermore, in a recent study by Liu et al. [52, 53], 580 genes related to five principal pathways (inflammation, metabolism, telomerase, DNA repair, and cognitive) and neurocognitive functions in 233 patients with glioma were analyzed. They demonstrated that 18 polymorphisms may be associated with processing speed and 12 with executive functions. These results are important since the identification of genetic markers could become useful for predicting the performance of cognitive deficits related to cancer and its treatments.

## 6. Conclusion

It is very difficult to draw firm conclusions on the basis of the studies considered. In the reported studies, research designs are different, as well as the number of patients, the disease diagnosis, the tests used, and the adopted rehabilitation training.

As shown by the various studies, a cognitive rehabilitation program involves a large commitment in terms of time and compliance of both patient and relatives who accompany him. Because of this, many patients often cannot participate because of the long distance from the treatment center or because no one can accompany them or because they tire of the program over time. Despite these problems and those arising from differences in the assessment of cognitive functions and implementation of the rehabilitation program, the reviewed studies reach the conclusion that a rehabilitation intervention can be very useful in patients with glioma and, more generally, in patients with other brain tumors [9, 10]. Moreover, despite being very challenging, patients and caregivers seem to be satisfied and believe that the training was very useful.

In conclusion, it seems that the studies conducted up to now bring back the encouraging results on the application of rehabilitation techniques on deficit in cognitive function in patients with cerebral tumor. Patients and family members seem to be satisfied with the results and it seems that patients benefit from greater autonomy in everyday life and that family members have a lesser burden. It is important that future studies in exploring this topic be structured in an appropriate manner, by providing a control group and a randomized selection of patients in the groups. Knowledge of specific genetic mutations involved in the development of cognitive deficits may be directed to personalized cognitive rehabilitation programs and to minimizing the appearance of deficit.

## Figures and Tables

**Table 1:** Principal characteristics of study population.

Investigators (year, name)	Design	*n*	Population
Maschio et al. (2015) [13]	Observational study	16	4 HGG, 2 GBM, 5 LGG, 2 meningioma (MEN), 3 metastasis (MET), related epilepsy Age > 18 y

Yang et al. (2014) [14]	Randomized control trial	38	5 glioblastoma (GBM), 2 astrocytoma (AST), 10 MEN, 6 MET, 15 other

Zucchella et al. (2013) [15]	Randomized control trial	58	25 HGG, 7 LGG, 16 MEN, 5 other

Hassler et al. (2010) [16]	Clinical trial	11	6 GBM, 5 LGG

Gehring et al. (2009) [17]	Randomized control trial	140	117 LGG, 23 HGG Age > 18 y

Locke et al. (2008) [18]	Randomized control trial	19 patient-caregiver dyads	13 HGG, 6 LGG before or upon initiation of radiation therapy Age > 18 y; presence of 1 caregiver

Sherer et al. (1997) [19]	Retrospective study	13	1 GBM, 9 LGG, 1 embryonal choriocarcinoma, 1 pineoblastoma, 1 anaplastic ependymoma

Principal characteristics of studies taken into consideration are in chronological order of publication.

HGG: high-grade glioma; GBM: glioblastoma; MEN: meningioma; MET: metastasis; AST: astrocytoma; LGG: low-grade glioma.

**Table 2:** Description of test used and domains assessed.

Investigators (year, name)	Functions assessed	Tests used for assessment
Maschio et al. (2015) [13]	Global neurocognitive performance, attention, executive functions, abstract reasoning, visuospatial abilities, long-term visuospatial memory, short-term and long-term auditory-verbal memory, language.	MMSE [20]; TMT A and TMT B [21]; FAB [22]; PM 47 [23]; ROCF-copy [24]; ROCF recall [24]; clock drawing test [25]; span forward and backward [26]; recall of disyllabic words [27]; 15 Rey-Osterreith word list [28]; immediate recall; 15 Rey-Osterreith word list; delayed recall [28]; story recall [29]; phonemic and semantic fluency tests [30]

Yang et al. (2014) [14]	Continuous concentration on visual and auditory items, selective attention, verbal and spatial memory, visual-motor coordination, activities of daily living.	Computerized neuropsychological tests (CNTs) [31]; Korean version of MMSE (K-MMSE) [32]; K-MBI [33]

Zucchella et al. (2013) [15]	Language disturbance, global cognitive functioning, verbal and spatial immediate memory span, verbal memory, immediate and delayed recall, nonverbal reasoning, frontal functionality, simple speed processing, complex attention, visual selective attention, visuoconstructional abilities, verbal fluency.	Before starting training: the ENPA [34] Neuropsychological battery: MMSE [20]; digit span [26]; Corsi's test [26]; RAVLT and logical memory [28, 29]; PM47 [35]; FAB [22]; TMT A and TMT B [36]; attentive matrices [27]; ROCF-copy [24]

Hassler et al. (2010) [16]	Verbal memory, attention, visual-motor speed, executive functions, verbal fluency.	TMT A [37]; TMT B [37]; HVLT [38]; COWA [39]

Gehring et al. (2009) [17]	Attention, verbal memory, executive functions, motivations, general cognitive functions.	Screening tests: DART [33]; DMT [20]; SCWT [26, 29]; LDST [28]; MST [35]; VVLT direct and delayed recall [22]; CST [36]; CF animals [27] Neuropsychological tests for the evaluation of intervention effects: SCWT [26, 29]; LDST [28]; MST [35]; TEA [24]; VVLT direct and delayed recall [22]; CST [36]; LF [34]; CF animals and professions [27]; BADS [40, 41]; TEA [24]; CFS [42]; burden (study-specific measure); CFQ [43]; SF-36 [44, 45]; MFI [46]; CIQ [47]

Locke et al. (2008) [18]	Immediate memory, visuoconstruction abilities, language, attention, delayed memory.	The Compensation Techniques Questionnaire; study-specific poststudy feedback questionnaire; FACT–BR16 [40]; LASA scale of QOL [43, 44]; CQOLC [47, 48]; MPAI-4 [41, 42]; R-BANS [45, 46]; POMS [49]; BFI [50]

Sherer et al. (1997) [19]	Verbal and visual memory, intellectual, executive functions, language, motor, visual perception, mood. Also productivity status and level of independence were assessed.	Not specified

Description of the tests used in the studies taken into consideration and the principal domains assessed.

Studies are presented in chronological order of publication.

MMSE: Minimental State Examination [20]; TMT A TMT B: trail making test A+B [36]; FAB: Frontal Assessment Battery [22]; PM 47: Raven Progressive Matrices 47 [35]; ROCF-copy: Rey-Osterrieth Complex figure, copy [24]; ROC Freecall: Rey-Osterrieth Complex figure, recall [24]; CNTs: computerized neuropsychological tests [31]; K-MMSE: Korean version of MMSE [32]; K-MBI: Korean version of Modified Barthel Index [33]; ENPA-Esame Neuropsicologico per l'Afasia: The Neuropsychological Exam for Aphasia [34]; RAVLT: Rey Auditory-Verbal Learning Test [28, 29]; HVLT: Hopkins Verbal Learning Test [38]; COWA: Controlled Oral Word Association Test [39]; DART: Dutch Adult Reading Test; DMT: Drie-Minuten-Toets (Three-Minute Test); SCWT: Stroop Color-Word Test; LDST: Letter Digit Substitution Test; MST: Memory Scanning Test; VVLT: Visual Verbal Learning Test, direct and delayed recall; CST: Concept Shifting Test; CF: Category Fluency animals, from the GIT; DS: Digit Span; TEA: Test of Everyday Attention; LF: Letter Fluency, CT professions, from the GIT; BADS: Behavioural Assessment of the Dysexecutive Syndrome; CFS: Cognitive Functioning Scale from the MOS, burden (study-specific measure); CFQ: Cognitive Failure Questionnaire; SF-36: Short-Form 36 from the MOS; MFI: Multidimensional Fatigue Inventory; CIQ: Community Integration Questionnaire; FACT-BR1: Functional Assessment of Cancer Therapy, brain version [40]; MPAI-4: the Mayo-Portland Adaptability Inventory-4 [41, 42]; R-BANS: Repeatable Battery for the Assessment of Neuropsychological Status [45, 46]; LASA: Linear Analogue Self-Assessment scale of QOL [43, 44]; CQOLC: Caregiver QOL Index-Cancer [47, 48]; POMS: Profile of Mood States [49]; BFI: Brief Fatigue Inventory [50].

**Table 3:** Program of rehabilitation, timing of training, and domains trained.

Investigators (year, name)	Type of program	Timing	Cognitive functions improved
Maschio et al. (2015) [13]	TNP software (neuropsychological training software) was the program used for training. Training exercises included word and image lists, selection and recognition of targeted stimuli, spatial orientation, and phonological abilities.	Once a week for ten weeks.	Memory, attention, visuospatial functions, language, and reasoning.

Yang et al. (2014) [14]	Intervention group: virtual reality training based on real-time and three-dimensional environment and computer-assisted cognitive rehabilitation together. Control group: only computer-assisted cognitive rehabilitation.	Intervention group: VR training was done 3 times a week for 30 minutes and computer cognitive rehabilitation 2 times a week for 30 minutes. Control group: received computer-assisted cognitive rehabilitation 5 times a week for 30 minutes. Each group was trained for a total of 4 weeks.	Memory (spatial memory, recognition, sequential recall, verbal recall, verbal categorization, verbal and nonverbal memory), attention (discrimination, visual perception, auditory perception, continuous attention, integration, emotional attention).

Zucchella et al. (2013) [15]	Intervention group: computer exercises guided by a neuropsychologist, training different cognitive functions. Types of software utilized for computerized exercises were “training di riabilitazione cognitiva” and “una palestra per la mente.” Both groups received usual medical and physiotherapy rehabilitative care.	Training was administered in one-hour sessions, four sessions per week, for 4 weeks (16 sessions in total).	Time and spatial orientation, visual attention, logical reasoning, memory, and executive functions.

Hassler et al. (2010) [16]	The program used in this study was the holistic mnemonic training program developed by Dr. Stengel. This training comprised the use of all the senses, emotions, and intellect of patients in exercises designed to develop skills in everyday life. Each session addressed, separately, all aspects of mental activity.	One session a week for 10 weeks. Each session lasted 90 minutes.	Perception, concentration, attention, memory, retentiveness, verbal memory, and creativity.

Gehring et al. (2009) [17]	Intervention group: cognitive retraining and compensation techniques. Compensation training consisted of six psychoeducational sessions that included both practical and tutorial lessons targeted at improving memory, attention, and executive functions. Regarding the retraining consisting of a specific computer program developed by the researchers (Concentration Car, C-Car), patients were asked also to complete the computerized homework. Three months after the end of the training, patients received a follow-up session by telephone aimed at strengthening certain aspects of the compensation training. Control group received usual care only and no cognitive rehabilitation training. At the end of the intervention group's training, the control group also received rehabilitation training.	The intervention group received cognitive training in two-hour sessions for six weeks.	Attention, memory, and executive function.

Locke et al. (2008) [18]	The rehabilitation program used provided both cognitive rehabilitation and problem-solving therapy. In interventions group, patients and caregiver learned to use a memory notebook for compensation of memory deficits and a positive problem-solving model useful in everyday life. Control group received standard medical care and no rehabilitation.	Six sessions of both cognitive rehabilitation and problem-solving therapy, over two weeks.	Memory, attention, and problem-solving skills.

Sherer et al. (1997) [19]	The goal of rehabilitation therapy was individual for each patient: return to work or increased community independence or return to school. Once a patient improved his/her functions at hospital, he/she was transitioned to an occupational setting therapy where he/she could perform skills similar to the desired vocational goal. At the end of the program, patients were helped to return to their desired productive activities.	The typical session day lasted 5 hours.	

Description of the rehabilitation programs used in studies taken into consideration, timing of training, and domains trained. Studies are presented in chronological order of publication.

**Table 4:** Major results.

Investigators (year, name)	Patients satisfactions	Cognitive results
Maschio et al. (2015) [13]	nd	After rehabilitation training: improved scores in span forward, long-term visuospatial memory, episodic memory, and phonetic fluency compared to the baseline. The same functions remained stable at 6-month follow-up.

Yang et al. (2014) [14]	nd	Study group: improvements in visual and auditory continuous performance scores, digit span and visual span tests, verbal and visual learning tests, TMT A scores, and MMSE. Control group: improvement in auditory continuous performance score, visual and verbal learning tests, forward visual span test, and MMSE.

Zucchella et al. (2013) [15]	nd	Study group: significant improvement in all neuropsychological tests. Control group: trend improvement but it was not statistically significant. A statistically significant difference between groups was only found in visual attention and verbal memory but not in logical-executive functioning.

Hassler et al. (2010) [16]	All patients were very satisfied with the training.	Evaluation of neurocognitive functions done before and after training demonstrated that an enhancement across all neurocognitive functions assessed was achieved. This improvement was statistically significant only in total learning scores of HVLT test.

Gehring et al. (2009) [17]	Patients reported that this type of training was very useful and strategies learned during the rehabilitation period were also used in everyday life.	Over time: significant differences between groups in objective evaluation of cognitive functions, in particular, on attention and verbal memory. In subjective evaluation, differences were found in CFS total score, CFQ total score, and burden. After the end of the training: no significant differences between groups. In subjective outcomes, the intervention group had better reported cognitive functioning. 6 months after the end of the treatment: scores of the study group differed significantly from control group for attention and verbal memory. In subjective outcomes, no differences were found.

Locke et al. (2008) [18]	88% of patients and caregivers think that the program could be “very helpful” or “somewhat helpful.”	88% of patients in study group used the study-specific strategies in the range of several times a week (minimum) to several times a day (maximum). At three-month follow-up, 50% patients used techniques in the range of several times a week (minimum) to several times a day (maximum) and 88% employed them but less frequently in time. It was not possible to know if the assessed cognitive functions had improved, remained stable, or deteriorated over time, because many of the patients did not show up for the follow-up evaluation.

Sherer et al. (1997) [19]	nd	Patients improved during the treatment period. This gain was generally maintained at follow-up 8 months after discharge. Patients had increased community, employment and financial independence, and quality of life.

Studies are presented in chronological order of publication.
